# Extended Wang sum and associated products

**DOI:** 10.1371/journal.pone.0276078

**Published:** 2022-10-17

**Authors:** Robert Reynolds, Allan Stauffer

**Affiliations:** Department of Mathematics and Statistics, York University, Toronto, ON, Canada; Hodeidah University, YEMEN

## Abstract

The Wang sum involving the exponential sums of Lerch’s Zeta functions is extended to the finite sum of the Huwitz-Lerch Zeta function to derive sums and products involving cosine and tangent trigonometric functions. The general theorem used to derive these sums and products is in the form of the finite sum over positive integers of the Hurwitz-Lerch Zeta function where the associated parameters are general complex numbers. New Hurwitz-Lerch Zeta function recurrence identities with consecutive neighbours are derived. Some finite sum and product formulae examples involving cosine, tangent and the product of cosine and tangent functions are also derived and evaluated.

## 1 Introduction

The theory of finite sums and products involving trigonometric functions has been studied by various authors over many years. Some studies conducted in this area were by Ganier et al. [1] where a generalization of a trigonometric identity involving Fibonacci numbers was provided. In the work of Sury [2], a certain polynomial identity was used to express Fibonacci and the Lucas numbers in terms of trigonometric functions. In the work of Chamberland [3], several product and sum identities were established with special cases involving Fibonacci and Lucas numbers. In the work by Wang [4], finite sums of the Hurwitz Zeta function were derived in terms of Bernoulli numbers and Bernoulli polynomials, where a special case led to the classical Eisenstein formula [5]. The work of Nakamura [6], considered the universality for linear combinations of Lerch Zeta functions. The works of Laurinčikas [7] and J. Steuding [8] treated universal Dirichlet series with the case that the compact sets $K_{l}$ are disjoint. The study of the Hurwitz-Lerch Zeta function has been conducted by Min-Jie et al. [9] where the authors investigated an extended form of a Beta function and the study of the convergence problem of the function. They considered the completely monotonic and log-convex properties of this generalized Beta function. In the work of Choi et al. [10], a new extension of the generalized Hurwitz-Lerch Zeta functions of two variables was introduced. In the work of Parmar et al. [11], further generalization of the extended Hurwitz-Lerch Zeta functions were developed. In the work of Parmar et al. [12] a generalized form of the extended Hurwitz-Lerch Zeta function was considered with an emphasis on obtaining classical properties which include various integral representations, a differential formula, Mellin transforms, and certain generating relations. In the work of Jangid et al. [13], the authors established the composition formulas for the pathway fractional integral operator connected with Hurwitz-Lerch Zeta function and extended Wright-Bessel function. Based on previously published literature there is an obvious use for finite sums and products involving special functions and trigonometric functions. In this paper we develop a general theorem which includes the finite sum of Hurwitz-Lerch Zeta functions expressed in terms of Hurwitz-Lerch Zeta functions. This theorem represents a linear combination of Hurwitz-Lerch Zeta functions which could have advantageous uses in analysis. The outline of this paper is three-fold. We first derive the contour integral representation in terms of a formula given in Prudnikov et al. [14]. Next we apply the contour integral method [15] to derive the Cauchy integral representation in terms of the Hurwitz-Lerch Zeta function. We finally derive some closed form cases and examples in terms of trigonometric functions and mathematical constants. Our preliminaries begin with deriving the main contour integral formula to be used in this paper. The formula is derived by applying the contour integral method of [15] to equation (4.4.7.11) in [14]. Let $k,a,m\in C,n\in Z^{+},Re\left(m+w\right)>0$ then

$$\begin{array}{c} \frac{1}{2\pi i}\int_{C}\sum_{p=0}^{n}2^{-p}a^{w}w^{-k-1}\text{tan}(2^{-p}\left(m+w\right))dw\\ =\frac{1}{2\pi i}\int_{C}a^{w}w^{-k-1}(2^{-n}\;\text{cot}(2^{-n}\left(m+w\right))-2\;\text{cot}\left(2\left(m+w\right)\right))dw. \end{array}$$ (1)

In this work we derive the expression for the Hurwitz-Lerch Zeta function in terms of the sum of two Hurwitz-Lerch Zeta functions given by

$$\begin{array}{l} \sum_{p=0}^{n}4^{1-p}{\left(i2^{1-p}\right)}^{k-1}\\ e^{im2^{1-p}}\Phi \left(-e^{i2^{1-p}m},-k,1-i2^{p-1}\;\text{log}(a)\right)\\ =2i\left(\frac{1}{2}i{\left(i2^{1-n}\right)}^{k+1}e^{im2^{1-n}}\Phi \left(e^{i2^{1-n}m},-k,1-i2^{n-1}\;\text{log}(a)\right)\right.\\ \left.+2^{2k+1}e^{\frac{1}{2}i(\pi k+8m)}\Phi \left(e^{4im},-k,1-\frac{1}{4}\;i\text{log}(a)\right)\right), \end{array}$$ (2)

where the variables *k*, *a*, *m* are general complex numbers and *n* is any natural number. This new expression is then used to derive special cases with potential connection to Fibonacci numbers. The derivations follow the method used by us in [15]. This method involves using a form of the generalized Cauchy’s integral formula given by

$$\begin{array}{c} \frac{y^{k}}{\Gamma (k+1)}=\frac{1}{2\pi i}\int_{C}\frac{e^{wy}}{w^{k+1}}dw, \end{array}$$ (3)

where y,w∈C and *C* is in general an open contour in the complex plane where the bilinear concomitant [15] has the same value at the end points of the contour. This method involves using a form of Eq (3) then multiplies both sides by a function, then takes the definite integral of both sides. This yields a definite integral in terms of a contour integral. Then we multiply both sides of Eq (3) by another function and take the infinite sum of both sides such that the contour integral of both equations are the same.

## 2 The Hurwitz-Lerch Zeta function

We use equation (1.11.3) in [16] where Φ(*z*, *s*, *v*) is the Hurwitz-Lerch Zeta function which is a generalization of the Hurwitz zeta *ζ*(*s*, *v*) and Polylogarithm functions *Li*_*n*_(*z*). The Hurwitz-Lerch Zeta function has a series representation given by

$$\begin{array}{c} \Phi \left(z,s,v\right)=\sum_{n=0}^{\infty }{\left(v+n\right)}^{-s}z^{n} \end{array}$$ (4)

where |*z*| < 1, *v* ≠ 0, −1, −2, −3,.., and is continued analytically by its integral representation given by

$$\begin{array}{c} \Phi \left(z,s,v\right)=\frac{1}{\Gamma (s)}\int_{0}^{\infty }\frac{t^{s-1}e^{-(v-1)t}}{e^{t}-z}dt \end{array}$$ (5)

where *Re*(*v*)>0, and either |*z*| ≤ 1, *z* ≠ 1, *Re*(*s*)>0, or *z* = 1, *Re*(*s*)>1.

## 3 Finite sum of the contour integral

We use the method in [15]. The cut and contour are in the first quadrant of the complex *w*-plane with 0 < *Re*(*w* + *m*)<1. The cut approaches the origin from the interior of the first quadrant and goes to infinity vertically and the contour goes round the origin with zero radius and is on opposite sides of the cut. Using a generalization of Cauchy’s integral formula (3) we first replace *y* → log(*a*) + *i*2^1−*p*^(*y* + 1) then multiply both sides by *i*2^1−*p*^(−1)^*y*^ *e*^*im*2^1−*p*^(*y* + 1)^ and take the infinite sums over *y* ∈ [0, ∞) and *p* ∈ [0, *n*] and simplify in terms of the Hurwitz-Lerch Zeta function to get

$$\begin{array}{c} \sum_{p=0}^{n}\frac{i2^{-p}({\text{log}}^{k}\left(a\right)-2{\left(i2^{1-p}\right)}^{k}e^{im2^{1-p}}\Phi (-e^{i2^{1-p}m},-k,1-i\;2^{p-1}\text{log}\left(a\right)))}{\Gamma (k+1)}\\ =\frac{1}{2\pi i}\sum_{y=0}^{\infty }\sum_{p=0}^{n}\int_{C}i2^{1-p}a^{w}w^{-k-1}e^{i(2^{1-p}\left(y+1\right)\left(m+w\right)+\pi y)}dw\\ =\frac{1}{2\pi i}\int_{C}\sum_{p=0}^{n}\sum_{y=0}^{\infty }i2^{1-p}a^{w}w^{-k-1}e^{i(2^{1-p}\left(y+1\right)\left(m+w\right)+\pi y)}dw\\ =\frac{1}{2\pi i}\int_{C}\sum_{p=0}^{n}2^{-p}a^{w}w^{-k-1}\text{tan}(2^{-p}\left(m+w\right))-i2^{-p}a^{w}w^{-k-1}dw\\ =\frac{1}{2\pi i}\int_{C}a^{w}w^{-k-1}(2^{-n}\text{cot}(2^{-n}\left(m+w\right))-2\text{cot}\left(2\left(m+w\right)\right))dw \end{array}$$ (6)

from equation (4.4.7.11) in [14] where $Im\left(m+w\right)>0,Re\left(m+w\right)>0,n\in Z^{+}$, in order for the sums to converge. We apply Tonelli’s theorem for multiple sums, see page 177 in [17] as the summand is of bounded measure over the space C×[0,n]×[0,∞).

### 3.1 The additional contour integral

We use the method in [15]. Using a generalization of Cauchy’s integral formula (3) we first replace *y* → log(*a*) and multiply both sides by −*i*2^−*p*^ simplify to get

$$\begin{array}{c} -\frac{i2^{-p}\;{\text{log}}^{k}\left(a\right)}{\Gamma (k+1)}=-\frac{1}{2\pi i}\int_{C}i2^{-p}a^{w}w^{-k-1}dw \end{array}$$ (7)

## 4 Infinite sum of the contour integral

### 4.1 Derivation of the first contour integral

We use the method in [15]. Using a generalization of Cauchy’s integral formula (3) we first replace *y* → log(*a*) + 4*i*(*y* + 1) then multiply both sides by 4*ie*^4*im*(*y* + 1)^ and take the infinite sums over *y* ∈ [0, ∞) and simplify in terms of the Hurwitz-Lerch Zeta function to get

$$\begin{array}{c} \frac{i4^{k+1}e^{\frac{1}{2}i\left(\pi k+8m\right)}\Phi (e^{4im},-k,1-\frac{1}{4}i\;\text{log}\left(a\right))}{\Gamma (k+1)}\\ =\frac{1}{2\pi i}\sum_{y=0}^{\infty }\int_{C}4ia^{w}w^{-k-1}e^{4i(y+1)(m+w)}dw\\ =\frac{1}{2\pi i}\int_{C}\sum_{y=0}^{\infty }4ia^{w}w^{-k-1}e^{4i(y+1)(m+w)}dw\\ =-\frac{1}{2\pi i}\int_{C}(2a^{w}w^{-k-1}\text{cot}\left(2\left(m+w\right)\right)+2ia^{w}w^{-k-1})dw \end{array}$$ (8)

from equation (1.232.1) in [18] where *Im*(*m* + *w*) > 0 in order for the sum to converge.

#### 4.1.1 The additional contour integral

We use the method in [15]. Using a generalization of Cauchy’s integral formula (3) we first replace *y* → log(*a*) and multiply both sides by −2*i* simplify to get

$$\begin{array}{c} -\frac{2i\;{\text{log}}^{k}\left(a\right)}{\Gamma (k+1)}=-\frac{1}{2\pi i}\int_{C}2ia^{w}w^{-k-1}dw \end{array}$$ (9)

### 4.2 Derivation of the second contour integral

We use the method in [15]. Using a generalization of Cauchy’s integral formula (3) we first replace *y* → log(*a*) + *i*2^1−*n*^(*y* + 1) then multiply both sides by −2^1−*n*^ *ie*^(2*ibm*(*y* + 1))^ and take the infinite sums over *y* ∈ [0, ∞) and simplify in terms of the Hurwitz-Lerch Zeta function to get

$$\begin{array}{c} -\frac{{\left(i2^{1-n}\right)}^{k+1}e^{im2^{1-n}}\Phi (e^{i2^{1-n}m},-k,1-i2^{n-1}\;\text{log}\left(a\right))}{\Gamma (k+1)}\\ =-\frac{1}{2\pi i}\sum_{y=0}^{\infty }\int_{C}i2^{1-n}a^{w}w^{-k-1}e^{i2^{1-n}\left(y+1\right)\left(m+w\right)}dw\\ =-\frac{1}{2\pi i}\int_{C}\sum_{y=0}^{\infty }i2^{1-n}a^{w}w^{-k-1}e^{i2^{1-n}\left(y+1\right)\left(m+w\right)}dw\\ =\frac{1}{2\pi i}\int_{C}2^{-n}a^{w}w^{-k-1}\text{cot}(2^{-n}\left(m+w\right))+i2^{-n}a^{w}w^{-k-1}dw \end{array}$$ (10)

from equation (1.232.1) in [18] where *Im*(*m* + *w*)>0 in order for the sum to converge.

#### 4.2.1 The additional contour integral

We use the method in [15]. Using a generalization of Cauchy’s integral formula (3) we first replace *y* → log(*a*) and multiply both sides by *i*2^−*n*^ simplify to get

$$\begin{array}{c} \frac{i2^{-n}{\text{log}}^{k}\left(a\right)}{\Gamma (k+1)}=\frac{1}{2\pi i}\int_{C}i2^{-n}a^{w}w^{-k-1}dw \end{array}$$ (11)

## 5 The finite sum of the generalized exponential Hurwitz-Lerch Zeta function

**Theorem 5.1.** *For all* k,a,m∈C,n∈N *then*,

$$\begin{array}{c} \sum_{p=0}^{n}4^{1-p}{\left(i2^{1-p}\right)}^{k-1}e^{im2^{1-p}}\Phi (-e^{i2^{1-p}m},-k,1-i2^{p-1}\;\text{log}\left(a\right))\\ =2i(\frac{1}{2}i{\left(i2^{1-n}\right)}^{k+1}e^{im2^{1-n}}\Phi (e^{i2^{1-n}m},-k,1-i2^{n-1}\;\text{log}\left(a\right))\\ +2^{2k+1}e^{\frac{1}{2}i\left(\pi k+8m\right)}\Phi (e^{4im},-k,1-\frac{1}{4}i\;\text{log}\left(a\right))) \end{array}$$ (12)

*Proof*. Observe that the addition of the right-hand sides of Eqs (6) and (7), is equal to the addition of the right-hand sides of Eqs (8), (9), (10) and (11) so we may equate the left-hand sides and simplify the Gamma function to yield the stated result.

**Example 5.2.** *The degenerate case*.

$$\begin{array}{c} \sum_{p=0}^{n}2^{-p}\text{tan}(m2^{-p})=2^{-n}\text{cot}(m2^{-n})+\text{tan}\left(m\right)-\text{cot}\left(m\right) \end{array}$$ (13)

*Proof*. Use Eq (12) and set *k* = 0 and simplify using entry (4) of Section (64:12) in [19].

## 6 Recurrence identity and products of trigonometric functions

In this section several product identities are established similar to the work on Fibonacci and Lucas numbers in [3] along with a new recurrence identity for the Hurwitz-Lerch Zeta function.

**Example 6.1.** *A recurrence identity with consecutive neighbours*.

$$\begin{array}{c} \Phi \left(z,s,a\right)=2^{1-s}z\Phi (z^{2},s,\frac{a+1}{2})+\Phi \left(-z,s,a\right) \end{array}$$ (14)

*Proof*. Use Eq (12) and set *n* = 0, *a* = *e*^*ai*^, *m* = −*i* log(−*z*), *k* = −*s* and smplify.

**Example 6.2**. *A recurrence identity with consecutive neighbours*.

$$\begin{array}{c} \Phi \left(z,s,a\right)=2^{1-2s}z(2^{s}\Phi (-z^{2},s,\frac{a+1}{2})+2z^{2}\Phi (z^{4},s,\frac{a+3}{4}))+\Phi \left(-z,s,a\right) \end{array}$$ (15)

*Proof*. Use Eq (12) and set *n* = 1, *a* = *e*^*ai*^, *m* = log(*z*)/*i*, *k* = −*s* and smplify.

**Example 6.3**. *A Finite Product Involving the Quotient Product of Cosine Functions*.

$$\begin{array}{c} \prod_{p=0}^{n}\frac{{\text{cos}}^{2}(2^{-1-p}x)\text{cos}(2^{-1-p}x)}{{\text{cos}}^{2}(2^{-2-p}x)\text{cos}(2^{-p}x)}=\frac{\text{tan}(2^{-2-n}x)\text{tan}\left(x\right)}{\text{tan}(2^{-1-n}x)\text{tan}(\frac{x}{2})} \end{array}$$ (16)

*Proof*. Use Eq (12) and set *k* = 1, *a* = 1, *m* = *x* and apply the method in section (8) in [20].

**Example 6.4**. *A Finite Product Involving the Quotient Product of Cosine Functions and Exponential Function. Plots of the right-hand side for real and complex ranges are produced*.

$$\begin{array}{c} \prod_{p=0}^{n}\frac{e^{i2^{-p}\left(m-q\right)}\text{cos}(2^{-p}m)}{\text{cos}(2^{-p}q)}=\frac{e^{i2^{-n}(-1+2^{1+n})\left(m-q\right)}(\text{sin}\left(2m\right)\text{sin}(2^{-n}q))}{\text{sin}(2^{-n}m)\text{sin}\left(2q\right)} \end{array}$$ (17)

*Proof*. Use Eq (12) and form a second equation by replacing *m* by *q* and take their difference and set *k* = −1, *a* = 1 and simplify using entry (3) of Section (64:12) in [19].

**Example 6.5**. *A Finite Product Involving The Exponential Of Trigonometric Functions*.

$$\begin{array}{c} \prod_{p=0}^{n}{\left(\frac{1+e^{i2^{-p}x}}{1+e^{i2^{1-p}x}}\right)}^{i\pi }e^{2^{-p}\;\text{tan}(2^{-p-1}x)\text{sec}(2^{-p}x)}\\ =2^{i\pi }{\left(\frac{\text{cos}(2^{-n-1}x)}{1+e^{2ix}}\right)}^{i\pi }\text{exp}(\text{csc}\left(x\right)\text{sec}\left(x\right)-2^{-n-1}(2\;\text{csc}(2^{-n}x)+\pi x)) \end{array}$$ (18)

*Proof*. Use Eq (12) and set *k* = 1, *a* = −1, *m* = *x* and apply the method in section (8) in [20].

**Example 6.6**. *A Finite Product Involving The Exponential Of Trigonometric Functions*.

$$\begin{array}{c} \prod_{p=0}^{n}{\left(\frac{1+e^{i2^{1-p}x}}{1+e^{i2^{-p}x}}\right)}^{-\frac{i\pi }{2}}e^{2^{-p}\;\text{tan}(2^{-p-1}x)\text{sec}(2^{-p}x)}\\ =2^{-\frac{i\pi }{2}}{\left(e^{2ix}\right)}^{i}{\left(e^{-i2^{-n-1}x}\right)}^{i\left(4+\pi \right)2^{n}}{\left((1+e^{i2^{-n}x})\text{sec}\left(x\right)\right)}^{\frac{i\pi }{2}}e^{-2^{-n}\text{csc}(2^{-n}x)+\text{tan}\left(x\right)+\text{cot}\left(x\right)} \end{array}$$ (19)

*Proof*. Use Eq (12) and set *k* = 1, *a* = *i*, *m* = *x* and apply the method in section (8) in [20].

**Example 6.7.** *A Finite Product Involving Quotient Cosine Functions*.

$$\begin{array}{c} \prod_{p=0}^{n}\frac{\text{cos}(2^{-1-p}x)}{\text{cos}(2^{-p}x)}=\frac{\text{cos}(2^{-1-n}x)}{\text{cos}(x)} \end{array}$$ (20)

*Proof*. Use Eq (19) and form a second equation by replacing *x* → −*x* and multiplying both and simplifying.

**Example 6.8**. *A Finite Product Involving Quotient Cosine Functions Raised To A Power*.

$$\begin{array}{c} \prod_{p=0}^{n}{\text{cos}}^{8}(2^{-p-3}x){\text{cos}}^{7}(2^{-p-1}x){\text{sec}}^{14}(2^{-p-2}x)\text{sec}(2^{-p}x)\\ ={\text{cos}}^{6}(\frac{x}{2}){\text{sec}}^{8}(\frac{x}{4})\text{sec}\left(x\right){\text{cos}}^{8}(2^{-n-3}x)\text{cos}(2^{-n-1}x){\text{sec}}^{6}(2^{-n-2}x) \end{array}$$ (21)

*Proof*. Use Eq (12) and set *k* = 2, *a* = 1, *m* = *x* and apply the method in section (8) in [20].

**Example 6.9.** *A Finite Sum Involving Tangent And Cosine Functions*.

$$\begin{array}{c} \sum_{p=0}^{n}8^{-p}(-4^{p}a^{2}+2\;{\text{sec}}^{2}(2^{-p}x))\text{tan}(2^{-p}x)\\ =\frac{2(a^{2}-8\;{\text{csc}}^{2}\left(2x\right))}{\text{tan}(2x)}+\frac{8^{-n}(-4^{n}a^{2}+2\;{\text{csc}}^{2}(2^{-n}x))}{\text{tan}(2^{-n}x)} \end{array}$$ (22)

*Proof*. Use Eq (12) and set *k* = 2, *m* = *x* and simplify using entry (4) in Section (64:12) in [19]. Next form a second equation by replacing *m* → −*m* and take their difference.

**Example 6.10**. *A Finite Sum Involving Tangent And Cosine Functions Raised To A Power*.

$$\begin{array}{c} \sum_{p=0}^{n}32^{-p}\;\text{tan}(2^{-p}x)(a^{4}16^{p}-4(3a^{2}4^{p}+2){\text{sec}}^{2}(2^{-p}x)+24\;{\text{sec}}^{4}(2^{-p}x))\\ =2^{-5n}\text{cot}(2^{-n}x)(a^{4}16^{n}-4(3a^{2}4^{n}+2){\text{csc}}^{2}(2^{-n}x)+24\;{\text{csc}}^{4}(2^{-n}x))\\ -\frac{\text{cos}\left(2x\right)(a^{4}\text{cos}\left(8x\right)+3\left(a-8\right)\left(a+8\right)a^{2}-4(a^{4}-48a^{2}-128)\text{cos}\left(4x\right)+2560)}{128\;{\text{sin}}^{5}\left(x\right){\text{cos}}^{5}\left(x\right)} \end{array}$$ (23)

*Proof*. Use Eq (12) and set *k* = 4, *m* = *x* and simplify using entry (4) in Section (64:12) in [19]. Next form a second equation by replacing *m* → −*m* and take their difference.

**Example 6.11**. *Finite Product Sum In Terms Of Trigonometric Functions*.

$$\begin{array}{c} \prod_{p=0}^{n}\frac{\text{cos}(2^{-1-p}x)}{\text{cos}(2^{-p}x)}\sum_{p=0}^{n}8^{-p}(-4^{p}a^{2}+\frac{2}{{\text{cos}}^{2}(2^{-p}x)})\text{tan}(2^{-p}x)\\ =\frac{\text{cos}(2^{-1-n}x)}{\text{cos}(x)}(2\;\text{cot}\left(2x\right)(a^{2}-8{\text{csc}}^{2}\left(2x\right))+8^{-n}\text{cot}(2^{-n}x)(-4^{n}a^{2}+2{\text{csc}}^{2}(2^{-n}x))) \end{array}$$ (24)

*Proof*. Use Eqs (20) and (22), multiply and simplify.

**Example 6.12**. *Finite Product Sum Involving The Golden Ratio*.

$$\begin{array}{c} \prod_{p=0}^{n}\text{cos}(\frac{1}{5}\pi 2^{-p-1})\text{sec}(\frac{\pi 2^{-p}}{5})\sum_{p=0}^{n}8^{-p}\;\text{tan}(\frac{\pi 2^{-p}}{5})(-4^{p}+2{\text{sec}}^{2}(\frac{\pi 2^{-p}}{5}))\\ =(\sqrt{5}-1)\text{cos}(\frac{1}{5}\pi 2^{-n-1})\\ \left(\frac{2\sqrt{1-\frac{2}{\sqrt{5}}}(\sqrt{5}-59)}{5+\sqrt{5}}-8^{-n}\text{cot}(\frac{\pi 2^{-n}}{5})(4^{n}-2\;{\text{csc}}^{2}(\frac{\pi 2^{-n}}{5}))\right) \end{array}$$ (25)

*Proof*. Use Eq (24) and set *a* = 1, *x* = *π*/5 and simplify.

**Example 6.13.** *Finite Sum Involving Hyperbolic Trigonometric Functions With Angular Coefficients*.

$$\begin{array}{c} \sum_{p=0}^{n}8^{-p}({\text{sech}}^{2}(2^{-p}x)(2^{p}\left(1-ax\right)-2x\text{tanh}(2^{-p}x))-a4^{p}(\text{tanh}(2^{-p}x)-1))\\ =8^{-n}(2^{n}(\left(1-ax\right){\text{csch}}^{2}(2^{-n}x)+a2^{n}(2^{n+1}-1))+\text{coth}(2^{-n}x)(a4^{n}-2x\;{\text{csch}}^{2}(2^{-n}x)))\\ -2a\text{coth}\left(2x\right)+4\;{\text{csch}}^{2}\left(2x\right)\left(ax+4x\;\text{coth}\left(2x\right)-1\right) \end{array}$$ (26)

*Proof*. Use Eq (12) and set *k* = 1, *m* = *x* and simplify. Next Multiply both sides by *x* and take the first partial derivative with respect to *x* and simplify. Next replace *a* → *e*^*ai*^, *x* → *xi* and simplify.

## 7 Integrating over a finite sum with factors containing the Polylogarithm function with powers of 2

We prepare now to meet an integral with an integrand expressed in terms of a finite sum of a special function. This evaluation has many interesting results namely the exponential Catalan’s constant *K*.

**Example 7.1.** *Finite Product Involving The Exponential Of Polylogarithm Function*.

$$\begin{array}{c} \prod_{p=0}^{n}\text{sec}(2^{-p}x)\text{cos}(\frac{2^{-p}x}{\beta })a^{-i2^{p-1}({\text{Li}}_{2}(-e^{i2^{1-p}x})-{\text{Li}}_{2}(-e^{\frac{i2^{1-p}x}{\beta }}))}\\ {\left((1+e^{i2^{1-p}x}){\left(1+e^{\frac{i2^{1-p}x}{\beta }}\right)}^{-1/\beta }\right)}^{x\;\text{log}(a)}\text{exp}(\frac{2^{-p}x(\text{tan}(\frac{2^{-p}x}{\beta })-\beta \;\text{tan}(2^{-p}x))}{\beta })\\ =\text{csc}\left(2x\right)\text{sin}(2^{-n}x)\text{sin}(\frac{2x}{\beta })\text{csc}(\frac{2^{-n}x}{\beta })\\ a^{\frac{x\;\text{log}(\frac{{\left(\frac{-1+e^{4ix}}{-1+e^{i2^{1-n}x}}\right)}^{\beta }(-1+e^{\frac{i2^{1-n}x}{\beta }})}{-1+e^{\frac{4ix}{\beta }}})}{\beta }-\frac{1}{4}i(2^{n+1}({\text{Li}}_{2}(e^{\frac{i2^{1-n}x}{\beta }})-{\text{Li}}_{2}(e^{i2^{1-n}x}))-{\text{Li}}_{2}(e^{\frac{4ix}{\beta }})+{\text{Li}}_{2}(e^{4ix}))}\\ \text{exp}(\frac{x(2^{-n}\text{cot}(\frac{2^{-n}x}{\beta })+\beta (-2^{-n}\text{cot}(2^{-n}x)-\text{tan}\left(x\right)+\text{cot}\left(x\right))+\text{tan}(\frac{x}{\beta })-\text{cot}(\frac{x}{\beta }))}{\beta }) \end{array}$$ (27)

*Proof*. Use Eq (12) and set *k* = 1, *m* = *x* and simplify. Next multiply both sides by *x* and take the definite integral over *x* ∈ [*x*, *x*/*β*] and simplify. Next take the exponential of both sides and simplify.

**Example 7.2.** *A Finite Product In Terms Of Catalan’s Constant K*.

$$\begin{array}{c} \prod_{p=0}^{n}{\left(\frac{1+e^{i\pi 2^{-p-2}}}{\sqrt{1+e^{i\pi 2^{-p-3}}}}\right)}^{\pi /8}\text{cos}(\pi 2^{-p-4})\text{sec}(\pi 2^{-p-3})\\ \text{exp}(\pi 2^{-p-4}(\text{tan}(\pi 2^{-p-4})-2\;\text{tan}(\pi 2^{-p-3}))+i2^{p-1}({\text{Li}}_{2}(-e^{i2^{-p-3}\pi })-{\text{Li}}_{2}(-e^{i2^{-p-2}\pi })))\\ =2^{\frac{24+\pi }{16}}\text{exp}(\frac{K}{4}+\frac{i\pi^{2}}{192}-\frac{1}{8}\pi \;\text{tan}(\frac{\pi }{8})+2^{-4-n}\pi \;\text{tan}(2^{-4-n}\pi ))\\ \text{exp}(\frac{1}{4}i{\text{Li}}_{2}(\sqrt[{4}]{-1})-i2^{-1+n}{\text{Li}}_{2}(e^{i2^{-3-n}\pi })+i2^{-1+n}{\text{Li}}_{2}(e^{i2^{-2-n}\pi }))\\ {\left(-\frac{\left(1-i\right)+\sqrt{2}}{2(-1+e^{i2^{-3-n}\pi }){\left(1+e^{i2^{-3-n}\pi }\right)}^{2}}\right)}^{\pi /16}\text{cos}(2^{-4-n}\pi )\text{sin}(\frac{\pi }{8}) \end{array}$$ (28)

*Proof*. Use Eq (27) and set *a* = *e*, *x* = *π*/8, *β* = 2 and simplify.

## 8 Conclusion

In this paper, we have presented a method for a new finite sum involving the Hurwitz-Lerch Zeta function along with some interesting sums and products using contour integration. The results presented were numerically verified for both real and imaginary and complex values of the parameters in the integrals using Mathematica by Wolfram.
